# Collectanea Medica

**Published:** 1814-12

**Authors:** 


### COLLECTANEA MEDICA.

*On the Difference of Opinion concerning an Insect as the Cause of the Itch. From Dr. Adams's Remarks on Dr. Bateman's Synopsis.*

I WAS much pleased to see the attention paid in the Synopsis to names and distinctions among the vulgar, p. 67 and 204, notes. This is also remarked in the text, under the article Itch, in which the vulgar names are neatly Latinized. On this occasion, I wish the author had availed himself of information from the same sources from which he derived his names. Few of the Irish labourers who apply for relief at the Carey-street Dispensary, would be found ignorant of the *flesh-worm*, of the difference between the disease it excites, and the *itch;* and even of the different manner in which each may be cured. The experiments made in Madeira, and confirmed in Ireland, have lately been repeated at Paris; but

 whether whether the vulgar were consulted, does not appear. In the Synopsis, we are referred to Abinzoar of the 12th, and others of the 15th and 16th centuries. Without questioning the accuracy of either, let me remark, that Ambrose Paré, about the last date, gives a very accurate account of these insects, and says, the patient may be cured by extracting them; after which he advises stavesacre, aloes, or salt, to be rubbed on the parts. Sauvages and Pleuck both advise extracting the insect. Can this be the disease in which Swammerdam, Canton, Baker, Heberden, and Hunter, never could discover an insect with the best microscopes?

It must be admitted, that since philosophers interfered in this question, it has been involved in much obscurity. Bonomo took half his lesson from the galley-slaves; and of his crude materials, Dr. Mead published a mutilated account in the Philosophical Transactions. From that time, philosophers and physicians have been much puzzled; some having seen the insect, which never could be detected by others, who searched for it with the best microscopes, in the most marked cases of itch. Sauvages, who distinguishes the two diseases in his octavo edition, confounds them in his later quarto edition. Dr. Willan, who was full of candour, was aware of these difficulties, and thought he could reconcile them, by finding that Sennertus, Mercurialis, and some other writers of their date, had described *pruritus* or *prurigo* as the frequent forerunner of scabies. His words are, when persons affected with the above disease neglect washing, "the eruption grows inveterate, and at length, changing its form, often terminates in the itch. Pustules arise among the papulæ, some filled with lymph, others with pus. The acarus scabiei begins to breed in the furrows of the skin, and the disease becomes contagious." This was published before I had given him the history of the disease, and shown him the insect, of which he procured me a drawing by the late Dr. Shaw. Had my friend Willan lived to complete his work, I have no doubt that he would have corrected this passage, and done justice to the sources of his information, as he never failed to do on other occasions.

In the Synopsis, there are two attempts to reconcile the difficulty. The first is, by approving Sauvages' arrangement, in making a separate species of S. Vermicularis; the other is, "by supposing that the breeding of these acari in a scabious skin, is a rare and casual circumstance, like the individual instance of a minute pulex in prurigo, observed by Dr. Willan; and that the contagious property of scabies exists in the fluid, and not in the transference of insects."

Does the expression "generated in some species of scabies only," mean that scabies is necessary for the generation of such an insect, or that there is a species of itch occasioned by the acarus cyro? The latter seems the meaning of Sauvages; but, were it as Dr. Bateman afterwards expresses himself, "a casual circumstance," it could not be "like the individual instance of pulex mentioned by Dr. Willan." We have then only to inquire, whether they will breed breed under the cuticle of a skin free from scabies? and whether the disease may be cured by extracting them? Both these facts have been experimentally ascertained. The conclusion is, that an eruptive disease, independent of the itch, is excited by an insect called in Ireland the flesh worm; in France, the çyron; in Portugal, ouçāõ; once well known in England by the name of wheal worm, and still sometimes occurring. That the disease which it excites, from some resemblance, from situation, and from yielding to the same remedies, may be mistaken for itch; but that the insect has never been discovered when searched for in the most marked cases of true itch.

In the Synopsis, we have four varieties of scabies. These four consist of the true itch only, and another disease, which I have called the *herpes pauperum*. This is the Scabies Cachectica of the Synopsis, a term which, if contagion were not attached to the genus, would be unexceptionable. It has been noticed by Sir John Pringle, by Dr. Gillespie, and by many other writers on camp and prison diseases. It occurs in confined nurseries, if change of air is not introduced, but without extending to other parts of the house. I have seen it in two or three misguided youths, who have returned to their parents, after a temporary absence, in the lowest order of society: in these instances, it has never been communicated to any of the family.* I have called the disease Herpes, from its disposition to spread along the skin. This etymology of the word is admitted in the Synopsis (from ερπειν *serpere.*) Yet, in the same work, "the appellation is limited to a vesicular disease, which, in most of its forms, passes through a regular course. Such is particularly the case with one "*local* variety," which, we are told, p. 233, "was not noticed by Dr. Willan." It is incidentally mentioned under a different name in Observations on Morbid Poisons, 2d edit. p. 94, and is most minutely described by Mr. Royston, in the Medical and Physical Journal, vol. xxiii. p. 446.

---

* See also Epidemics, chap. 1. From the last-mentioned circumstances, I was led to suppose, that the disease is only infectious in that kind of air in which it is generated.

If the reader is unacquainted with these distinctions, and has not leisure or inclination to refer to the arguments by which they are inforced, I must request him to give me credit for them. One illustration, however, occurs from this passage in the Synopsis. "The most ordinary cause of scabies, we are told, p. 196, is contagion."—"It seems, however, to originate in crowded, close, and uncleanly houses."—"When the contagion has been introduced, however, into families where every attention to cleanliness is enforced, it frequently spreads to all the individuals, children and adults," &c. All this is correct, if the genus *Scabies* is to include diseases arising from different causes, and different in their character, and even different in their mode of cure. But, in my opinion, it would be more correct to say, *Itch and Herpes pauperum* are both common in close and uncleanly houses—the former must be introduced into, but the latter may be generated in, such places, and both are communicable in such places. The former, when introduced into a clean family, may be communicated before the parties are aware of it; and will only yield to certain local remedies. The latter is never generated in such families, nor communicable when introduced; and is curable without the local remedies necessary in the former.

### *Etymology of the word Scrofula, and Definition of the Disease.*

Having mentioned the word Scrofula, after professing to avoid all technical expressions, I might perhaps excuse myself, by saying, that the word is sufficiently vernacularized; but unless it were as well understood, this would scarcely be a sufficient apology. The learned may require an etymology: this would be extremely difficult, in a word, the orthography of which is not well ascertained, some spelling it with the f, and others with the ph. The former derive the term from *Scrofa*, the Latin word for a sow that has frequently littered. Even here there is a doubt concerning the application of the etymology, some ascribing it to the frequency with which pigs are affected, and others to the multiplication of certain tumours, similar to a large litter of pigs. Most authors say, that it is a disease of the glands of a particular species; yet parts are often said to be affected by it in which none of these glands are discovered, as the bones, the lungs, and membranes of the eyes and nose. It is said to be hereditary, but it is only so in predisposition, always requiring some exciting cause. These causes are all of them such as produce a disposition to local disease, and lessen the restorative powers of the constitution, or of the diseased part. The most general of these is cold. Hence it has been remarked, that among the inhabitants of the colder parts of our island, there are few families who are not scrofulous; but this is only saying, that the exciting cause is constant among them, that therefore no individual of any family can entirely escape, who has the slightest predisposition to it. The truth is, that the inhabitants of warm climates are much more predisposed to scrofula, as is proved by the effect produced on them when exposed to the cause. Negroes in cold climates rarely escape; and the children sent by their parents from the south for education, often suffer the effects of a disease never before seen by the family.

Cold, however, is not the only exciting cause. Poor diet, bad clothing, and all the other attendants of poverty, or privations from any other cause in the most favourable climate, will produce a similar effect, but less aggravated than when increased by the continual operation of cold. The late Dr. Heberden, whom I so often mention, and always with pleasure, speaks of the unwholesomeness of diet or situation as an exciting cause. The inhabitants of Rheims are said to have been very much afflicted with scrofula, when they used no other water but from their wells; and to have been relieved by the introduction of the water of a neighbouring river. Is it not highly probable, that the meliorated condition of the inhabitants which enabled them to afford the expence of such aqueducts, might also, by proving the means of better diet, clothing, and lodging, have contributed to their exemption from scrofula? Every community, as it is poor, is subject to cutaneous and other local diseases, which are ill-conditioned and inveterate, in proportion as the means of relief are less in their power. It is universally admitted in Great Britain, that the inhabitants are more free from these complaints, as they are become richer. But the uncertainty of our climate will always expose those who have a

 strong strong susceptibility, because we never can be sufficiently prepared for changes of temperature, like the inhabitants of more settled, though colder, regions.

Scrofula is very well described by the common expression of bad flesh to heal, so that any one may become scrofulous by a low state of health, induced from any cause; but some constitutions, in every state of health, show a less aptitude for healing wounds than others. If, in these subjects, inflammation is excited in any part, there will be always danger of consequent abscess, and the healing process will be slow. In young subjects, the glands are the most liable to inflammation, probably on account of their increased action at the period of growth, in order to model the form to its various changes. Hence, the glands of the neck, by their exposure to cold, are the most liable to become scrofulous. Though the consequent scars, from their unsightliness, are usually considered as the strongest proofs of a scrofulous constitution, yet, as the causes which induced the inflammation of those glands have ceased, there is no reason to apprehend any subsequent symptoms, without the access of some external cause. White swellings, and a variety of other complaints, are very properly enumerated as scrofulous, yet they more frequently appear in those who have escaped the early symptoms. The terror of scrofula is much increased by its sometimes affecting the lungs; but the most frequent form of consumption, we have already seen, is neither hereditary, nor connected with scrofula. In the same manner, the delicate skin and high complexion are often described as marks of a scrofulous constitution; but the fallacy of this is proved, by the frequency with which the disease attacks emigrants from the southern regions. The high complexion is, in this country, the usual attendant on the family consumption, and marks an irritability very easily affected by external causes; it differs, however, from scrofula, inasmuch as the ulcers rarely become indolent.

On the whole, then, I should define scrofula to be that constitution in which local disease is excited by the slightest causes, and in which the restorative power is the most feeble; admitting that such a constitutional predisposition, like every other, is often hereditary, but that the disease itself may, for the most part, be prevented or cured, by avoiding the exciting cause.—*Dr. Adams on Hereditary Diseases.*

---

*Memoir upon the compound and smooth or simple Eyes of Insects, and on the Manner in which these two Species of Eyes concur in Vision.* By M. Marcel de Serres, Professor of the Sciences in the Imperial University.

(*Continued from p.* 403.)

The tunic of the cornea is generally black in the *coleopteræ:* it is the same in those which want the choroid and the mucous varnish. The colour of this tunic is on the contrary much more variable among the *orthopteræ*, and sometimes one and the same eye presents it of two different shades, as we see in the *locusta lilifolia:* lastly, in certain species the tunic of the cornea presents stripes stripes variously coloured, and traced with the greatest regularity. These stripes are parallel in the *grullus lineola:* some are of a blackish brown, and others of a yellowish grey, the clear colour of which dips into that of the rest of the eye. It must be remarked, that frequently these shades are effaced after death, on account of the alterability of the tunic of the cornea. There are even species in which the tunic of the cornea is as it were marbled; then it exhibits various shades in different parts of the eye.

As to the tunic of the smooth or simple eyes, it is seldom black in the *orthopteræ*, and its colours are always uniform in the same eye; although they are much variegated, when we examine a great number of species. The most common colours are white, red, or pale green.

A great number of *hemipteræ* also present the tunic of their cornea of colours pretty much variegated; but its brown or rather black tints are much more common than in the *orthopteræ*. As we find among the latter, several *hemipteræ* present the tunic of the cornea of their smooth eyes, either whitish or of the most agreeable red, as is observed in the common grasshopper. Lastly, it appears to me, that almost all the *hemipteræ* which live in water have the tunic of their cornea completely black; perhaps it is the same with all the *coleopteræ* which have the same way of living.

The *lepidopteræ*, furnished with the finest colours, have also the tunic of the cornea of shades greatly variegated. Nevertheless, among all those which fly at sunset or during the night only, we observe that the tunic of their cornea is generally black. To conclude: the case is the same with several species of diurnal butterflies: for instance, the *papilio podalirius* has the tunic of the cornea completely black; and notwithstanding the sombre hue of this colour, the varnish of the choroid is very thick in this species. In the *papilio atalanta* the tunic of the cornea is of a clear brown; thus the black point resulting from the aperture through which the optic nerve passes, is seen on the outside of the eye. It must be observed, however, that as this circular aperture is formed in this species by vesicular tracheæ, and these tracheæ being a little separated from each other, the black point which is seen generally at the exterior of the eye, seems surrounded by other black points, which present a peculiar form, all having a smaller extent than the central point. In the *papilio cardui*, the tunic of the cornea is of a brilliant green, but much clearer in the lower part of the eye than in the upper, on account of the luminous rays less easily reaching them. This tunic is thin, whereas the varnish of the choroid and the choroid itself are, on the contrary, thick. We also observe externally on the eye the central black point, and several others produced by the same cause with those of the eye of the *papilio atalanta*. Only these blackish points are more irregular in their form than those of the *papilio atalanta*.

The larvæ of the *lepidopteræ* have only smooth eyes situated generally on the sides of the head. They are variable in colour and number. Thus we observe eight in the silkworm, and six only in the caterpillar. The former are black, and the latter transparent.

In

In the *neuropteræ*, the tunic of the cornea is also very much varied, and its colours vary much in various species. In the *libellula vulgaris*, the compound eyes exhibit all their upper portion of a reddish brown, and all the lower of a yellowish green. On carefully removing the cornea, it is easily seen that the difference in the two colours is owing to the diversity of colour in the tunic of the cornea. It seems even that this tunic is thicker in the brown part of the eye than in the green. We shall also remark, that the upper facets of the cornea are greater than the lower, which is rather singular. If we remove the cornea, we distinctly perceive the optic nerves passing through the choroid and its varnish, membranes which are easily distinguished from their being of a deep black.

The circular trachea surrounds the retina, and its ramifications pierce with the optic nerves all the other membranes, dispersing themselves *ad infinitum* under the cornea. This disposition is so evident, that we may easily conceive how Swammerdam thought the cornea was formed by the meeting of these tracheæ. In other respects the eye of this *libellula* presents nothing particular, with the exception of the enlargement or great extent of the retina.

I have also dissected the eyes of certain larvæ of *agrion* and *aeshma*, but have observed nothing very different. These larvæ seemed to want the circular trachea, the place of which is supplied by an infinity of other tracheæ, which spread in a great number around the optic nerve, which is very broad and thick in these larvæ. I never observed that the eye had any particular conformation in the species which habitually frequent the water; and this remark is important, because the case is very different in the other animals. The form of the cornea also presents differences in the species in the aquatic insects, as well as in those which live habitually in the air.

If the tunic of the cornea presents shades much varied in the *lepidopteræ* and *neuropteræ*, these shades are not less varied in the *hymenopteræ*, in which we sometimes see a grey, a green, a yellow, and even all the shades of black. Certain bees, and particularly the *violacea*, present this tunic of the most opaque black. Generally the *hymenopteræ* have their cornea very thick, and it is not difficult to remove it by thin slices. Sometimes this cornea is surrounded by hairs, and rarely do they issue from the cornea itself. In a great number of species, the circular trachea is totally wanting; but its place is always supplied by other and smaller tracheæ which surround the optic nerve, and in general these tracheæ are extremely multiplied.

What has been said with respect to the eyes of the *hymenopteræ* is applicable to those of the *dipteræ*, which have the tunic of the cornea with the most variegated shades. In a certain number of species these shades are very brilliant, and the *tabanus* as well as the *musca* hold a distinguished part in this respect.

Certain species of *syrphus* present, at the exterior of the eye, two semicircular stripes deeper than the general tint. These stripes are produced as usual by a deeper shade of the tunic of the cornea in this part of the eye; but what renders this disposition remarkable is, that there are numerous hairs precisely in this very part of the compound eye, and which originate from the cornea itself.

The eyes of the *apteræ* are in general of dark colours, and not very large. These insects seem therefore not to be much favoured in respect to the organ of sight, and probably their way of life does not require acute vision.

It is remarkable that the insects which live in water have in general their eyes dim and opaque externally, nearly like those which exist in dark places. Thus the nymphs of the *libellulæ* in passing to the condition of a perfect insect assume brilliant and transparent eyes, whereas previous to their last metamorphosis these eyes were dull and without lustre. This observation has not escaped the sagacity of Reaumur in his History of Insects.

In the descriptions which we have given of the situation and form of the compound and simple eyes of insects, it will be remarked that the former disposition is less subject to vary than the latter. This consideration might have arisen *à priori,* since no form can exhibit differences in any order of animals, without other variations following in the parts which surround those whose disposition changes. The only very remarkable example which we can mention of the variation in the position of the compound eyes, is that of the *diopsis ichneumonea.* This insect exhibits its eyes situated nearly like those of the *mòllusci:* thus they are placed at the extremity of a long pedicle, or a kind of contractile tentacular point, but which can at least follow all the motions of the head. We may say, on observing this singular organization, that this insect has its eyes at the extremity of a telescope; but, as we have not seen it alive, nor dissected it, it would be difficult to decide how far this idea is correct.

As to the descriptions which we shall presently give of the relation which exists between the size of the bodies and that of the compound eyes, they prove that the *dipteræ*, the *hymenopteræ, lepidopteræ*, and *neuropteræ*, have very large eyes: it even seems that there are few animals equally favoured. As a great number of *coleopteræ* and *hemipteræ* have also very large eyes in comparison to their bodies, we may say that this size is very general in all insects. The two solitary examples of the *phasmæ* and *scolopendræ* ought not to prevent us from concluding, that, of all the animals, insects have the largest eyes in comparison with the size of their bodies.

§ II. *Of the simple or smooth Eyes.* The number of simple eyes is far from being as constant as that of the compound eyes. Certain species present two; others four, six, and eight; but in general we observe three. This number is even pretty constant in families which have at once compound and smooth eyes. We rarely see exceptions to this arrangement; and the *blatta*, as well as certain *acheta*, are perhaps the only kinds which, having compound eyes, have only two simple eyes situated on the upper part of of the head. When there are three simple eyes, they are always arranged in the form of a triangle, so that there are two lateral, and one in the middle; their situation is also constantly on the summit of the head. Sometimes, however, the simple eyes are arranged two on the summit, and one in the middle of the front of the head. This arrangement holds in the *gryllus*, the *truxalis*, the *acrydium*, the *locusta*, and the *gryllotalpa*.

The *empusa*, which have upon the summit of the head a small triangular elongation, cannot, on account of this organisation, see objects with their eye in the middle.

The general form of the simple eyes is very variable: it seems, however, that in general that of the lateral eyes is elongated and elliptical, whilst that of the middle one is round: there are also many exceptions in this respect, since the *acheta* and the *gryllotalpa* present that in the middle of a very much elongated oval, of which the greatest diameter is transversal.

The smooth or simple eye is formed of an external hard transparent membrane, convex externally, and concave internally. This membrane, enveloping the eye, necessarily determines its form: it is not composed, like that of the compound eyes, of an infinite number of facets, but rather of a membrane of a single piece, and on which no divisions are perceptible. We may consider this first membrane as a cornea, on account of its transparency, a transparency which we observe even at the exterior of the eye, on account of the little colour of the varnish applied on the membranes situated under it.

After having removed the cornea, we find a viscous tunic more or less thick, and of which the colour also undergoes considerable variations. Thus this tunic or varnish presents the most opposite colours: it is almost always black in the *hymenopteræ*, whereas it is whitish in the *orthopteræ*. In the caterpillar, this varnish is frequently black, yellow, or red, and sometimes it is of the most beautiful brilliant green. This viscous tunic has been regarded by Swammerdam as a kind of uvea, although it seems very like the tunic which covers the cornea in compound eyes.

We ought not, however, to decide that the tunic of the cornea of the simple eyes is distinct from the varnish of the choroid. This appears probable notwithstanding, since in certain species the external colour of the simple eyes is not the same with that of the varnish with which the choroid is covered. Thus much is certain, that the cornea of the simple eyes is always coated internally with a kind of varnish, the colour of which appears at the exterior of the eye on account of the transparency of the cornea. The coating surrounds the optic nerve, which proceeds into the concavity of the cornea after having passed through the choroid aud its varnish. Swammerdam very properly remarks, that the smooth eyes of insects receive nerves which are furnished to them by the cerebriform ganglion lodged in the head. Lyonnet, whose researches announce much sagacity, has also observed that the simple eyes of the willow caterpillar are composed posed of a cornea and a choroid traversed by the extremity of the optic nerve.

The optic nerves of the simple eyes issue immediately from the brain when these eyes are removed far enough from each other; but, when, on the contrary, they are close together, as observed in the caterpillar and a great number of larvæ, the optic nerves are only divisions of a larger nerve which issues directly from the brain.

Lyonnet has described this arrangement accurately, and the figure which he gives of it, Plate XVIII. No. 6, is very correct. Then there exists a peculiar membrane in the form of a funnel, to which the six branches of the optic nerve are attached, and this membrane ends at the place where the nerve itself divides into these six branches. We do not know positively, if this arrangement exists in the spiders: we only know that it does not hold in the scorpions. Besides, externally the eyes of the spiders and of the scorpions have the same structure and figure with the simple eyes of insects.

Immediately after the optic nerve and the tunic of the cornea, we observe a peculiar membrane, which we shall consider as a kind of choroid. It exhibits, however, this difference from the choroid of the compound eyes, that it has no varnish so distinct; and finally, its breadth is always greater than the circumference of the cornea itself. This membrane, coloured frequently in red or black, is also sometimes colourless; and lastly, its whiteness is so dull that it is easy to distinguish it from the tracheæ. The thickness of this membrane is great enough to make it resist even a long maceration.

In the species in which the simple eyes are whitish, we see this membrane cloathed with a coating of colours varying with the colours of the tunic which covers the choroid. This arrangement is even very perceptible at the exterior of the eye.

It would seem that this choroid is particularly very thick and white in such species as have their cornea concave and as if sunk. This arrangement must have taken place on account of the divergency undergone by the rays of light in arriving on a concave and transparent surface, their direction even being parallel, a divergency which is such that we may very well conceive that the eye would be too much affected by it, if a black membrane had also absorbed a certain number of luminous rays. A white membrane, sending back on the contrary all the rays which it receives, may augment the excitability of the optic nerve, and thus contribute to render the vision more distinct. Although this species of choroid presents in certain circumstances a very great whiteness, it is nevertheless always easy to distinguish it from the tracheæ, the only vessels with which it can be confounded. For, as little as we are in the habit of seeing the organs of insects, we can easily ascertain the tracheæ from their azure colour; and besides, by dissecting them in water (the most advantageous way of acting in these experiments), we see the tracheæ rise above the surface of the liquid ; and from that moment we can no longer have the smallest doubt, for the tracheæ are the only organs which have so little specific gravity.

This choroid, the membranes of which are close and very thick, seems formed by a cellular texture of very close meshes, over which a heap of tracheæ are distributed. It is therefore underneath the cornea that the optic nerve is situated, which issuing from the anterior faces of the brain proceeds to the middle of the eye. It does not appear that this optic nerve has any swelling at its base, or that a kind of retina is formed analogous to that of the compound eyes. In some species it appears to me that this nerve becomes broader towards its extremity, *i. e.* at the point where it corresponds to the cornea. I shall not, however, assert this positively, for it is possible that this dilatation may depend on the contraction produced by the section of the nerve. As I have not dissected these organs in the living subject, (which ought always to be done for the sake of precision,) I have not been able to verify, whether this dilatation depended on the sensibility of the nervous organ. However the case may be, the optic nerve before reaching the cornea traverses the muscles of the various parts of the head, sometimes enters a trachea, penetrates afterwards through the choroid and its varnish, and afterwards, when surrounded with the tunic of the cornea, corresponds to the internal surface of this membrane, on which it seems to lose itself.

As the cornea of the simple eyes is not, like that of the compound eyes, divided by numerous facets, it has not been necessary that the small nerves should give it a great number of small threads, but only that they should proceed to the point where the divergency of the rays of light should be the least considerable.

The small optic nerves which proceed to the simple eyes issue from the brain. As their position varies in regard to the other parts, they are sometimes the second, third, fourth, or fifth pair of nerves: this depends on their situation with respect to the various organs of the head, a situation which determines by what pair they are furnished. These nerves are directed always towards the simple eyes, being retained in their position either by pneumatic pouches, or by tracheæ, proceeding more or less obliquely according to their position with respect to the brain. They always terminate under the cornea, forming a kind of retina.

It results from this description, that the simple eyes are formed quite differently from the compound eyes. Thus the cornea of the former is all of one piece, whereas in the latter it is formed by the union of a great number of hexagonal facets. The tunic of the cornea does not exhibit any difference in the two kinds of eyes, except perhaps in point of thickness; but the varnish of the choroid is always less distinct in the simple than in the compound eyes. Finally, the choroid is not always black in the simple eyes, whereas it is uniformly in the the compound. Sometimes this membrane, instead of being black and opaque, is of a peculiar whiteness and lustre. To conclude: this membrane and its tunic are placed in the same way with respect to the other parts in both kinds of eyes, and their uses are also similar. The large circular trachea, which we observe in the compound eyes when they present a choroid, does not seem to exist in the simple eyes. In fact, the tracheæ or pneumatic pouches which belong to them are used in supporting the optic nerve, and perhaps also in forming a part of the choroid in that sort of eye. At least the tracheæ of insects seem to perform the functions of the blood-vessels which are observed on the choroid of the red-blooded animals.—*Phil. Mag.*

