# Errata

**Published:** 1815-01

**Authors:** 


					-P. 501, 1. 2, grullus, read gryllw.-?
And the reader is desired to observe, in this article relative to the eyes of
insects, thai where the order orthoptera is mentioned, the order hemiptera,
according to the Linnaean system, is understood.-

				

